# Cholecystectomy Damages Aging-Associated Intestinal Microbiota Construction

**DOI:** 10.3389/fmicb.2018.01402

**Published:** 2018-06-25

**Authors:** Wenxue Wang, Junfeng Wang, Julan Li, Pingping Yan, Yun Jin, Ruyi Zhang, Wei Yue, Qiang Guo, Jiawei Geng

**Affiliations:** ^1^Department of Infectious Diseases, The First People's Hospital of Yunnan Province, The Affiliated Hospital of Kunming University of Science and Technology, Kunming, China; ^2^Kunming University of Science and Technology, Kunming, China; ^3^Department of Hepatobiliary Surgery, The First People's Hospital of Yunnan Province, Kunming, China; ^4^Department of Gastroenterology, The First People's Hospital of Yunnan Province, Kunming, China

**Keywords:** cholecystectomy, intestinal bacteria, colorectal cancer, aging, bile acids

## Abstract

The intestinal microbiome is essential in humans to maintain physiological balance and nutrition metabolism. Laparoscopic cholecystectomy due to gallstone disease and cholecystitis can cause intestinal microbial dysbiosis, and following bile acid metabolism dysfunction, positions the patient at high risk of colorectal cancer. However, little is known regarding intestinal microbiota characteristics in post-cholecystectomy patients. Here, we compared the microbial composition of cholecystectomy patients with that of a healthy population. We determined that cholecystectomy eliminated aging-associated fecal commensal microbiota and further identified several bile acid metabolism-related bacteria as contributors of colorectal cancer incidence via elevation of secondary bile acids.

**Significance statement:**

We identified aging-associated fecal microbiota in a healthy population, which was lost in cholecystectomy patients. Absent intestinal bacteria, such as *Bacteroides*, were negatively related to secondary bile acids and may be a leading cause of colorectal cancer incidence in cholecystectomy patients. Our study provides novel insight into the connection between cholecystectomy-altered gut microbiota and colorectal carcinoma, which is of value for colorectal cancer diagnosis and management.

## Introduction

The development, aging, and coevolution of hosts and microorganisms can impact the composition of gut commensal microbiota. Changes in composition can, in turn, reflect healthy, or unhealthy conditions in local and distant organs and provide a reliable diagnostic and therapeutic tool for human diseases (Qin et al., 2012, 2014; Zeller et al., 2014). On the one hand, intestinal immune and epithelial cells synthesize antibacterial peptides to establish the gut microbiota (Goldszmid and Trinchieri, 2012; Mowat and Agace, 2014; Hancock et al., 2016). On the other hand, certain gut microbiome species can elicit chronic inflammation and reactive oxygen species (ROS)-mediated genotoxicity or secrete DNA-damaging toxins to promote colorectal cancer (Wu et al., 2009; McCoy et al., 2013; Schwabe and Jobin, 2013; Sears and Garrett, 2014; Boleij et al., 2015). For example, colorectal cancer-associated *Fusobacterium nucleatum*, colibactin-producing *Escherichia coli*, and *Bacteroides fragilis* can induce carcinogenesis-promoting activation of WNT-β-catenin signaling (Cuevas-Ramos et al., 2010; Goodwin et al., 2011; Rubinstein et al., 2013), with antibiotic management shown to lower the incidence of colon cancer (Chen et al., 2008; Yoshimoto et al., 2013; Moss, 2017). We previously studied spatial heterogeneity and co-occurrence patterns of intestinal microbes, which were found to have a close relationship with colorectal cancer and hepatic encephalopathy (Geng et al., 2013, 2014; Zhang et al., 2013, 2014).

Cholecystectomies alter bile flow into the intestine and bidirectional interactions between bile acids (BAs) and intestinal microbiota, thereby increasing bacterial degradation of bile acids into fecal secondary bile acids (Malagelada et al., 1973; Hepner et al., 1974). Abnormal proximal colonic absorption of fecal secondary bile acids is a signal of right-sided colon cancer (Cook et al., 1940; Hill et al., 1975; Linos et al., 1981). It has been reported that post-cholecystectomy patients show considerable changes in bowel habits and increased loss of bile acids from the intestine (Sauter et al., 2002). Gallbladder-synthesized surfactant protein D selectively binds to gut commensal bacteria and plays a key role in intestinal susceptibility to dextran sulfate sodium-induced colitis, with previous study validating the inter-organ connection of the gallbladder with intestinal immune homeostasis (Sarashina-Kida et al., 2017). Cohort studies have also reported symptomatic gallstones and cholecystectomy to be associated with higher occurrences of gastric (Fall et al., 2007; Chen et al., 2014), small intestine (Johansen et al., 1996; Goldacre et al., 2012), and colon cancers (Fall et al., 2007; Goldacre et al., 2012; Chen et al., 2014; Zhang et al., 2017). Previous research has also indicated a risk of colorectal cancer within the first 5 years in post-cholecystectomy patients (Chen et al., 2014). However, the correlation between gut microbiota composition with post-cholecystectomy time remains to be uncovered.

Intestinal epithelial cells sense bile acid through farnesoid X receptor (FXR) and G protein-coupled bile acid receptor 1 (TGR5) and can influence disease occurrence and therapy (Iida et al., 2013; Zheng et al., 2013). For instance, bile acids trigger TGR5 signaling to balance the production of pro-inflammatory and anti-inflammatory cytokines in intestinal mucosa. During this process, IL-10 differentiates T cells into immunosuppressive Treg cells, whereas IL-12 differentiates T cells into inflammation-promoting TH1 cells (Littman and Rudensky, 2010; Kamada et al., 2013). Abnormally high levels of secondary bile acids can give rise to oxidative damage to DNA, inflammation, activation of NF-κB, and increased cell proliferation of the colonic epithelium through TGR5 (Hofmann et al., 1987; Wang et al., 2011), and are thus deemed to be colorectal cancer-promoting factors (Bernstein et al., 2005, 2011). Intestinal microbes produce enzymes to metabolize primary bile acids into secondary bile acids, and thus may be connected with the incidence of colorectal cancer (Chiang, 2009; Gérard et al., 2014; Wahlström et al., 2016).

In this study, we identified changes in the abundance of fecal microbiota in post-cholecystectomy patients compared with a healthy population. As the healthy group exhibited aging-associated intestinal microbiota, decreased diversity in the microbial community were investigated in the post-cholecystectomy group, that may lead to increase of secondary bile acids and following promotion of colorectal cancer. Our work provides novel insight into the connection between cholecystectomy-altered gut microbiota and colorectal carcinoma.

## Materials and methods

### Ethics statement

All study protocols and procedures were approved by the Medical Ethics Board of the First People's Hospital of Yunnan Province, China, and were carried out in accordance with all relevant provincial, national, and international guidelines, including the Declaration of Helsinki. Written informed consent was obtained from all participants prior to their inclusion in the study.

### Sample collection

The outcome measure of cholecystectomy patients included no bile duct injury, wound infection, retained gallstones, abscess formation, and/or stenosis of the bile duct, 2 years after a cholecystectomy. Sample size was calculated by the following formula:

$$\text{n}=\left\lceil \frac{\left({\text{u}}_{\alpha }+{\text{u}}_{\beta }\right)}{\delta /\sigma }\right\rceil +\frac{1}{2}{\text{u}}_{\alpha^{2}}$$

α = 0.05, and type II error (false negative) β = 0.1. Correspondingly, table of *u*-value indicated u_α_ = 1.645, u_β_ = 1.282. The required number of cases is ≥13. Therefore, our study employed 15 samples for each subunit of healthy volunteers and 20 samples for each subunit of cholecystectomy patients to satisfy sample size requirement. The healthy volunteers and cholecystectomy patients were appraised and recruited by annual physical examination for healthy control. The healthy volunteers are cholecystectomy patient's spouses, brothers, sisters, or parents. Antibiotic-administrated healthy volunteers and patients in recent 3 months, alcohol drinker, and smoker were excluded.

All fecal samples were collected in sterilized tubes and immediately preserved at −80°C for further analysis. The 75 healthy volunteer samples (25 males, 50 females) were divided into five groups (H1, 20–29 years old; H2, 30–39 years old; H3, 40–49 years old; H4, 50–59 years old; H5, over 60 years old, 15 samples for each group). The 60 fecal samples from 60 post-cholecystectomy patients (30–59 years old) were divided into three groups (D1, 5–9 years; D2, 10–14 years; D3, over 15 years post-cholecystectomy, 20 samples for each group).

### DNA/RNA extraction and 16S rRNA amplicon sequencing

Genomic DNA extraction was carried out using the QIAamp DNA Mini Kit (Qiagen, Germany). The 16S rRNA gene (V3–V4 region) amplicon sequencing was carried out with the 16S Metagenomic Sequencing Library Preparation protocol developed by Illumina (San Diego, California, USA). Briefly, 50 ng of bacterial DNA was amplified using primers targeting the V3–V4 variable region of the 16S rRNA gene. The 16S amplicon PCR forward primer (V3 region) was: 5′ TCGTCGGCAGCGTCAGATGTGTATAAGAGACAGCCTACGGGNGGCWGCAG; and the 16S amplicon PCR reverse primer (V4 region) was: 5′ GTCTCGTGGGCTCGGAGATGTGTATAAGAGACAGGACTACHVGGGTATCTAATCC. DNA was sequenced at Sangon Biotech (Shanghai, China) on a MiSeq sequencing instrument (Illumina, USA). The read length was centralized on 440–540 bp for the paired sequencing run.

### Statistical analysis

Sequencing reads were quality filtered, operational taxonomic unit (OTU) clustered. The QIIME software (Qiime 1.8.0, ggtern 2.1.1) was used to further analyze sequencing data (Caporaso et al., 2010). Data were excluded when read lengths were < 200 bp. 16S reference sequences were classified at species-level with 83% bootstrap support.

Pearson's correlations or associations between the most abundant 100 OTUs were calculated. Statistic *P*-values were corrected using the FDR method of the R.igraph package. Pearson's correlation was transformed into links between two OTUs in the OTU co-occurrence network.

PICRUSt (Phylogenetic Investigation of Communities by Reconstruction of Unobserved States), a computational approach to predict the functional composition of a metagenome using marker gene data and a database of reference genomes, uses an extended ancestral-state reconstruction algorithm to predict which gene families are present and then combines gene families to estimate the composite metagenome. Using 16S information, PICRUSt recaptures key findings from the Human Microbiome Project and accurately predicts the abundance of gene families in host-associated and environmental communities, with quantifiable uncertainty. Bray-Curtis distances were calculated using as implemented in the QIIME “beta_diversity.py” script (Langille et al., 2013).

Distance matrices were calculated using unweighted UniFrac principal component analysis (PCA). Community hierarchical clustering was carried out to evaluate community variation at the species level. Multiple sample comparisons were performed using Tukey-Kramer one-way analysis of variance (ANOVA). Significance was assumed for adjusted *P*-values of ≤ 0.05.

## Data availability statements

The sequencing data for this study can be found in the Sequence Read Archive (SRA) with accession SRP149836 (https://www.ncbi.nlm.nih.gov/sra).

## Results

### Healthy population showed aging-associated intestinal microbiota

**Figure 1:**
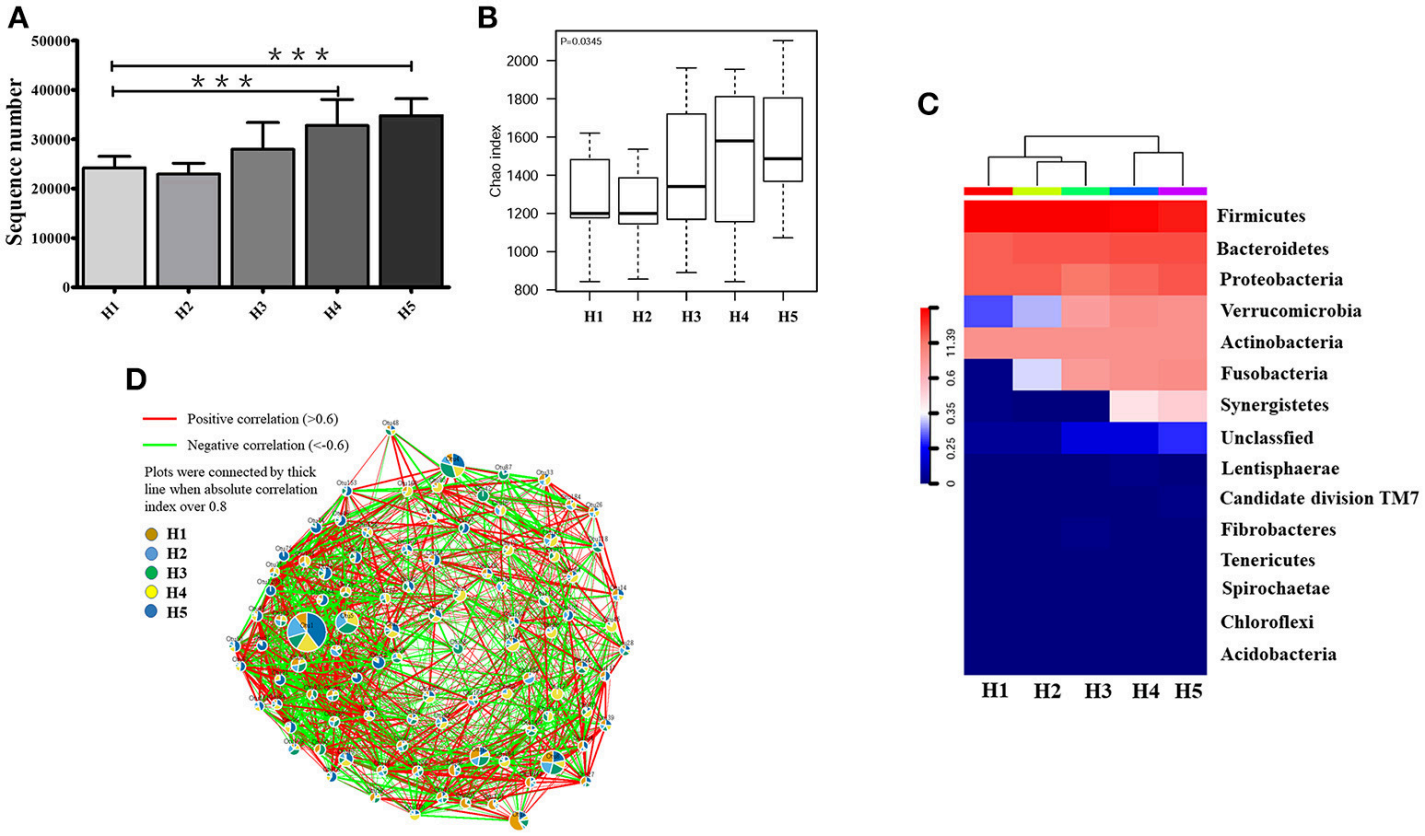
Alteration in aging-associated intestinal microbiota. **(A)** The number of OTUs identified by high-quality 16S ribosomal RNA sequencing in fecal samples from healthy volunteers of different ages. ^***^P < 0.001. **(B)** Alpha diversity of fecal microbiota from healthy volunteers of different ages. **(C)** Community hierarchical clustering analysis of healthy volunteers of different ages at the phylum level. Red color with high value (11.39) means high abundance of bacteria. Blue color with low value (0) means low abundance of bacteria. **(D)** Co-network plots of operational taxonomic units (OTUs) based on the fecal abundance of each OTU in the healthy population. Size of each pie correlates to the mean abundance of each OTU across all samples. H1, 20–29; H2, 30–39; H3, 40–49; H4, 50–59; H5, over 60 years old.

The high-quality 16S ribosomal RNA sequencing results showed a growing microbial community with aging. The operational taxonomic units (OTUs) identified by 16S rRNA sequencing was significantly higher in the older H4 and H5 groups than in the younger H1 group (*P* < 0.001; Figure 1A). Consistent with this result, the Chao index of the microbial community gradually increased in an age-dependent manner (*P* = 0.0345; Figure 1B). The heatmap of dominant phyla, which included Firmicutes, Bacteroidetes, Proteobacteria, Verrucomicrobia, Actinobacteria, Fusobacteria, and Synergistetes, also illustrated this aging-associated intestinal microbiota (Figure 1C).

In the oldest group (over 60 years old), abundances of operational taxonomic units including OTU1, OTU9, OTU46, OTU50, OTU57, OTU63, OTU74, OTU75, OTU91, OTU95, OTU86, OTU151, OTU153, OTU6581, and OTU12031 were significantly increased compared with that in the early stage, and showed strong positive correlation with each other. In contrast, OTU7, OTU8, OTU15, OTU27, OTU36, OTU104, OTU120, OTU201, and OTU6642 were greatly enriched and positively correlated with each other in the early stage (20–29 years old) (Figure 1D).

*Anaerotruncus, Akkermansia, Blautia, Faecalibacterium*, and *Parabacteroides* in the H1 group, *Akkermansia, Asteroleplasma, Faecalibacterium, Gardnerella*, and *Lachnospiraceae incertae sedis* in the H2 group, *Dorea, Faecalibacterium, Parabacteroides, Paraprevotella, Turicibacter*, and *Rothia* in the H3 group, and *Anaerotruncus, Blautia*, and *Lachnospiraceae incertae sedis* in the H4 group demonstrated significant differences in abundances compared with the older H5 group (*P* < 0.05; Table 1).

**Table 1:** Significantly age-varied microbial abundance of each age group compared with the H5 group in a healthy population at the genus level (H1, 20–29; H2, 30–39; H3, 40–49; H4, 50–59; H5, over 60 years old).

**Age stage (years old)**	**Abundance-different genus vs. H5**	**Difference between means**	***P*-value**	**95.0% CI**	
				**Lower**	**Upper**
Hl (20–29)	*Anaerotruncus*	−0.5856	0.008	−0.9968	−0.1743
	*Akkermansia*	−1.3082	0.031	−2.4812	−0.1352
	*Blautia*	1.3226	0.007	0.38804	2.25715
	*Faecalibacterium*	3.8194	0.013	0.88937	6.74948
	*Parabacteroides*	−0.3180	0.012	−0.5613	−0.0746
H2 (30–39)	*Akkermansia*	−1.3113	0.032	−2.4962	−0.1264
	*Asteroleplasma*	−0.0084	0.030	−0.0160	−0.0009
	*Faecalibacterium*	4.7384	0.010	1.26370	8.21328
	*Gardnerella*	−0.0029	0.023	−0.0053	−0.0004
	*Lachnospiraceae_Incertae Sedis*	8.0212	0.034	0.62869	15.4138
H3 (40–49)	*Dorea*	0.2769	0.026	0.03620	0.51768
	*Faecalibacterium*	4.1112	0.026	0.53656	7.68594
	*Parabacteroides*	−0.2850	0.020	−0.5199	−0.0500
	*Paraprevotelfa*	−0.4822	0.041	−0.9445	−0.0198
	*Turicibacter*	0.0309	0.012	0.00766	0.05417
	*Rothia*	0.0022	0.028	0.00026	0.00418
H4 (50–59)	*Anaerotruncus*	−0.4507	0.047	−0.8961	−0.0052
	*Blautia*	3.0990	0.000	1.48096	4.71719
	*Lachnospiraceae_Incertae Sedis*	7.4413	0.027	0.89285	13.9897
HS (≥60)	–	–	–	–	–

*Difference between means was calculated by H5 group mean minus younger group mean*.

Furthermore, co-network plots indicated that the older H5 group was extensively enriched with *Escherichia shigella, Bacteroides, Coprobacillus, Planomicrobium, Intestinimonas, Phyllobacterium, Coprobacter, Butyrivibrio, Cloacibacillus*, and *Shuttleworthia*, which were positively correlated with each other (Figure S1). Interestingly, phyla *Fusobacteria* and *Verrucomicrobia* and genera *Fusobacterium, Akkermansia*, and *Parabacteroides* were only correlated in the older H4 and H5 groups, and genera *Anaerotruncus* and *Paraprevotella* were only correlated in the H5 group (Figures S2A,B).

Further KEGG analysis suggested that the intestinal microbe framework may contribute to functional variation in their host in an aging-dependent manner (Figure S3), including variation in energy metabolism, N-Glycan biosynthesis, proximal tubule bicarbonate reclamation, RIG-I-like receptor signaling pathway, transporters, and biosynthesis of tropane, piperidine, and pyridine alkaloid (Tables S1A,B).

### Cholecystectomy decreased community diversity of intestinal microbiota

**Figure 2:**
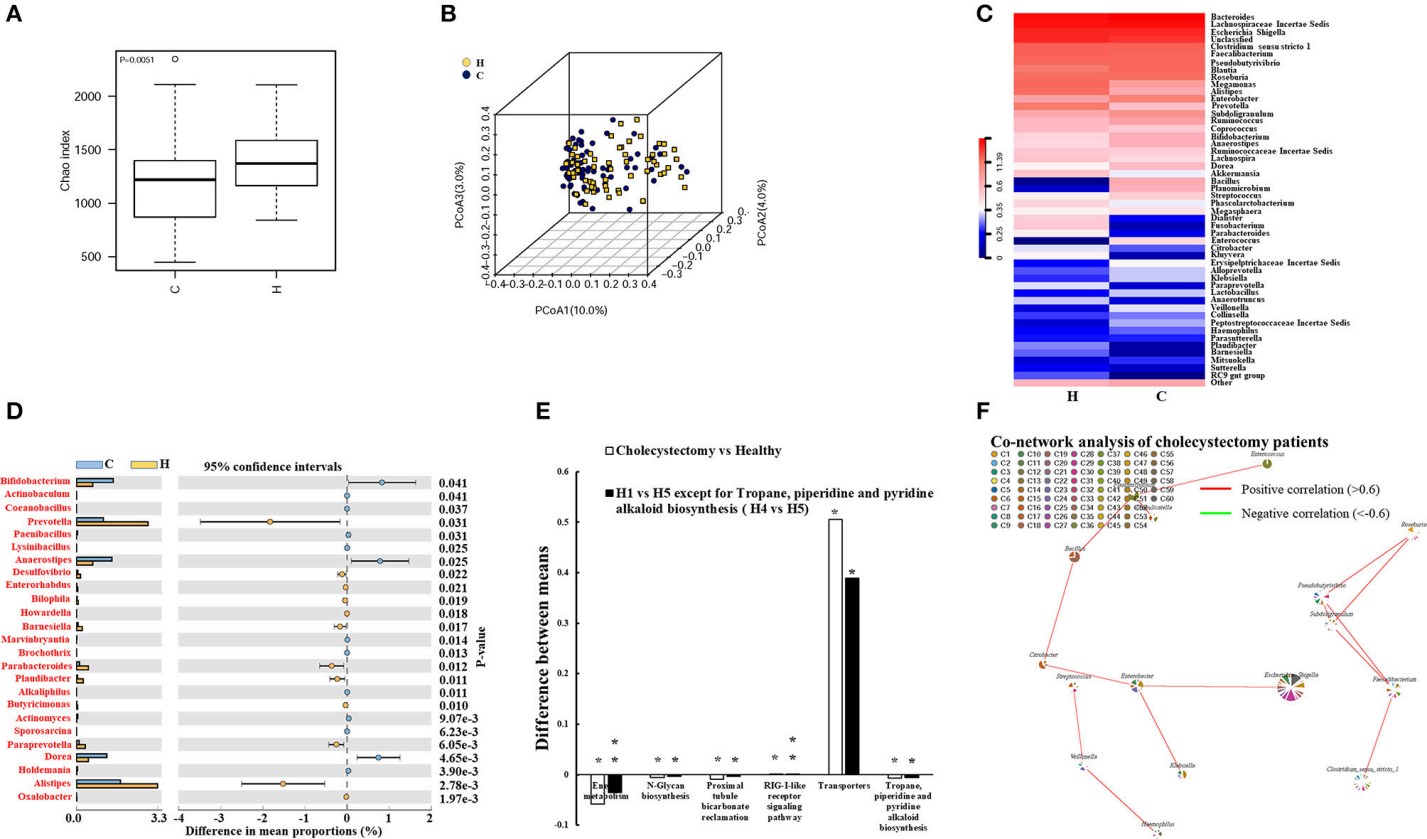
Alteration in cholecystectomy-induced intestinal microbiota. **(A)** Alpha diversity of fecal microbiota from cholecystectomy patients compared with the healthy population. **(B)** Unweighted UniFrac principal coordinates analysis (PCoA) of fecal microbiota from cholecystectomy patients and healthy controls. **(C)** Community hierarchical clustering analysis of healthy volunteers and cholecystectomy patients at the genus level. Red color with high value (11.39) means high abundance of bacteria. Blue color with low value (0) means low abundance of bacteria. **(D)** Significantly different abundance of fecal microbiota between healthy volunteers and cholecystectomy patients at the genus level. Genus co-network plots based on fecal abundance of bacteria in cholecystectomy patients **(E)**. **(F)** Size of each pie correlates to the mean abundance of each genus across all samples. Comparison of aging-altered function (H1 vs. H5 or H4 vs. H5) and cholecystectomy-altered function (cholecystectomy vs. healthy). ^*^P < 0.05; ^**^P < 0.01. H1, 20–29; H4, 50–59; H5, over 60 years old. H, healthy control group; C, cholecystectomy patient group.

We investigated the fecal microbiota in cholecystectomy patients and a healthy population. We observed significantly attenuated community richness in cholecystectomy patients (Figure 2A), despite the number of bacteria identified from sequencing was not significantly changed (Figure S4). Unweighted PCoA analysis revealed that the fecal microbe community in cholecystectomy patients had changed in PCoA axis (10%), see Figure 2B.

As shown in Table 1, higher *Anaerotruncus, Parabacteroides*, and *Paraprevotella* enrichment was observed in the older H5 group compared with that in the younger groups; however, cholecystectomy patients did not exhibit the same enrichment, with the abundances of these three genera demonstrating significant decreases compared with the abundances in the healthy population. In addition, the abundances of *Prevotella, Desulfovibrio, Barnesiella, Paludibacter*, and *Alistipes* all decreased, whereas those of *Bifidobacterium, Anaerostipes*, and *Dorea* all increased in the cholecystectomy patients (Figure 2C). Twenty-five genera with most significantly different abundance between healthy population and cholecystectomy patients were listed (Figures 2C,D).

*Escherichia coli, Fusobacterium nucleatum*, and *Bacteroides fragilis* are reported to play key roles in promoting colorectal cancer (Cuevas-Ramos et al., 2010; Kostic et al., 2012; Boleij et al., 2015). We revealed considerable increases in *E. coli TOP291* in cholecystectomy patients, thus providing potential evidence for cholecystectomy-induced colorectal cancer risk (Figure S5). The Environment for Tree Exploration (Python ete3 package) verified the sequence with strain level resolution (Jaime Huerta-Cepas et al., 2016).

Our results suggested that cholecystectomy patients may exhibit poor performance in aging-associated functions of the intestinal microbiota but tend to maintain their fecal microbiota at the early stage (Figure 2E). We also found other KEGG-predicted functions to be significantly different in cholecystectomy patients, including bacterial secretion system, biotin metabolism, carbon fixation pathways, and vitamin B6 metabolism (Tables S2A,B).

Even though the abundances of several genera were reduced, novel correlations among fecal microbiota were observed in the cholecystectomy patients. For example, *Pseudobutyrivibrio* and *Subdoligranulum* were positively correlated with *Faecalibacterium* and *Roseburia*. The correlative axis was also investigated among *Citrobacter, Bacillus, Planomicrobium*, and *Enterococcus* (Figure 2F).

### Attenuated aging-associated intestinal microbiota in cholecystectomy patients

**Figure 3:**
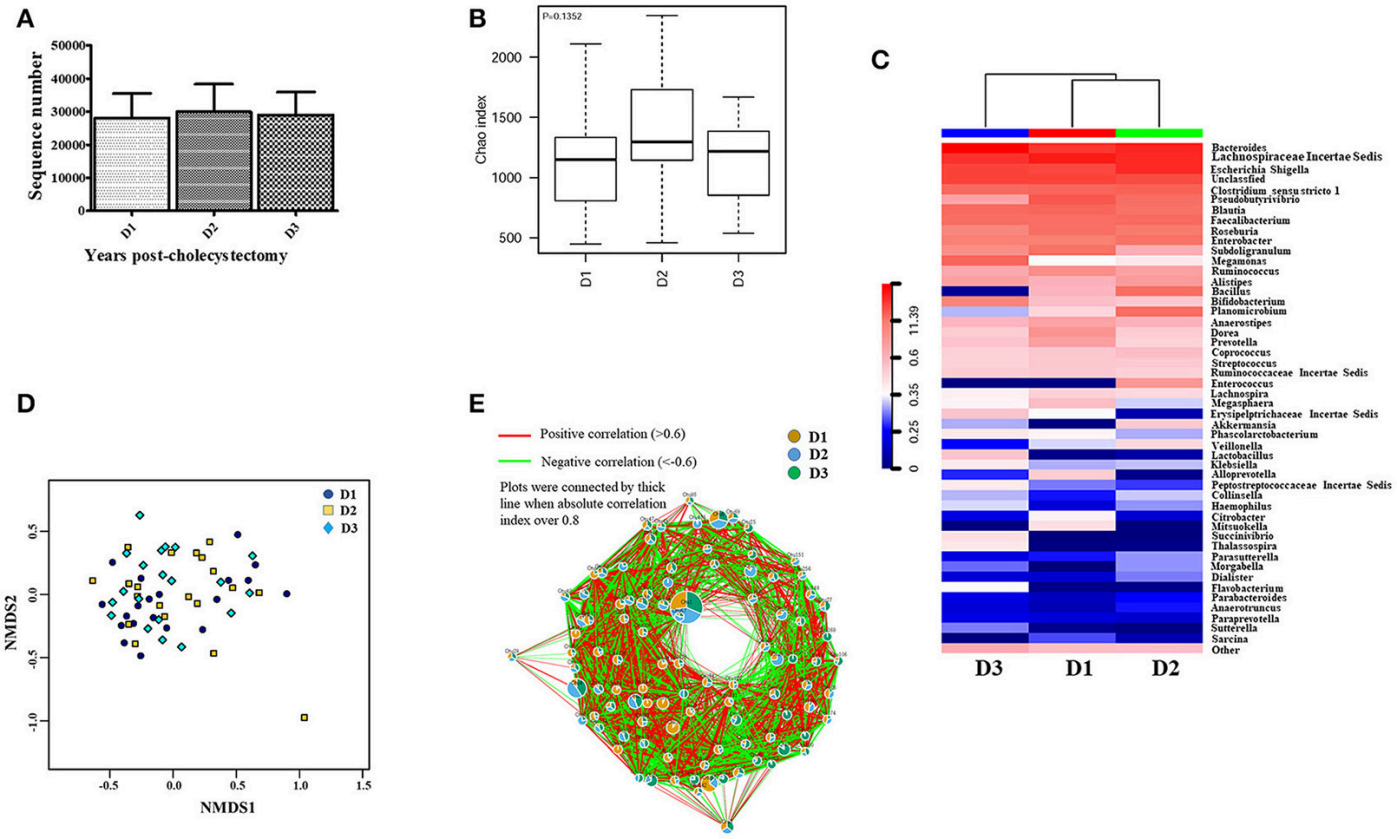
Fecal microbiota was not significantly altered with time post-cholecystectomy. **(A)** The number of OTUs identified by 16S ribosomal RNA sequencing in fecal samples from cholecystectomy patients at different time stage. **(B)** Alpha diversity of fecal microbiota from cholecystectomy patients at different time stage. **(C)** Community hierarchical clustering analysis of cholecystectomy patients at different time stage at the genus level. **(D)** Non-metric multidimensional scaling (NMDS) of fecal microbiota from cholecystectomy patients at different time stage. Red color with high value (11.39) means high abundance of bacteria. Blue color with low value (0) means low abundance of bacteria. **(E)** Co-network plots of operational taxonomic units (OTUs) based on the fecal abundance of each OTU in cholecystectomy patients at different time stage. Size of each pie correlates to the mean abundance of each OTU across all samples. D1, 5–9; D2, 10–14; D3, over 15 years after cholecystectomy.

The above results revealed that the aging-associated intestinal microbiota was lost in cholecystectomy patients; however, greater detail on intestinal microbiota construction after significant time post-cholecystectomy (decades) is required. Thus, we compared the identified OTUs number and alpha diversity of microbiota in patients 5–10 years, 10–15 years, and more than 15 years after cholecystectomy. Results showed that, regardless of time post-cholecystectomy, patients did not exhibit remarkable variation in fecal microbiota (Figures 3A,B). Although several genera, including *Pseudobutyrivibrio, Megamonas, Bacillus, Planomicrobium, Dorea*, and *Enterococcus*, changed in abundance (Figure 3C), only *Pseudobutyrivibrio, Metascardovia, Flavonifractor*, and *Selenomonas* showed significance (Table 2, *P* < 0.05).

**Table 2:** Significantly different microbial abundances in each post-cholecystectomy group compared with the D3 group at the genus level (D1, 5–9; D2, 10–14; D3, over 15 years after cholecystectomy).

**Post-cholecystectomy (years)**	**Age stage (years old)**	**Abundance-different genus vs. D3**	**Difference between means**	***P-*value**	**95.0% CI**	
					**Lower**	**Upper**
D1 (5–9)	30–39	*Pseudobutyrivibrio*	5.8740	0.010	1.5113	10.2367
		*Metascardovia*	0.0011	0.042	0.0000	0.0022
D2 (10–14)	40–49	*Flavonifractor*	−0.0030	0.036	−0.0058	−0.0002
		*Selenomonas*	0.0018	0.038	0.0001	0.0035
D3 (≥15)	50–59	–	–	–	–	–

*Difference between means was calculated by D3 group mean minus younger group mean*.

We applied non-metric multidimensional scaling (NMDS) to distinguish fecal microbiota over the three different times post-cholecystectomy; however, no evident profiles were observed (Figure 3D). Similar results were also displayed in the OTU (Figure 3E) and genus co-network analyses, with the dominant pies in the relevant figure divided almost equally into three portions (Figure S6). Although the KEGG Bray-Curtis tree suggested time-correlated variations in intestinal microbial function in cholecystectomy patients (Figure S7), including amoebiasis, bisphenol degradation, fructose, and mannose metabolism, and seleno-compound metabolism differentiation (Table S3), most diminished with age, contrary to the aging-associated intestinal microbiota.

## Discussion

The human intestine is inhabited by a diverse range of microbiota, including probiotic and enterotoxigenic bacteria (Jia et al., 2008; Nicholson et al., 2012). Inhabitants in the human intestine can strongly affect development, health, and disease, such as cancer, as well as responses to anticancer immunotherapy (Sommer and Bäckhed, 2013; Viaud et al., 2013; Sivan et al., 2015; Vétizou et al., 2015; Brennan and Garrett, 2016). Different microbes play various roles in cancer therapy. For example, *E. coli* and *Comamonas* conversely modulate a host's response to camptothecin (CPT) and 5-fluoro-20-deoxyuridine (FUDR) management (García-González et al., 2017). We found the higher abundances of genera *Bifidobacterium, Anaerostipes*, and *Dorea* increased in the cholecystectomy patients compared with healthy population, even the patients and healthy population had same age range (Table S4). Some of these microbes may metabolize toxins to promote colorectal cancer in cholecystectomy patients. Interestingly, we found that the abundance of *E. coli TOP291* increased significantly in cholecystectomy patients, suggesting cholecystectomy-related colorectal cancer could be resistant to CPT and FUDR therapies. Certainly, metabolite analysis is needed to confirm what kind of toxins is produced by these microbes in cholecystectomy patients.

Mice raised under germ-free conditions show increased fecal levels of conjugated bile acids and reduce secondary bile acids in the intestines of mice, with similar bile acid profiling also revealed in inflammatory bowel disease patients (Brestoff and Artis, 2013; Duboc et al., 2013). Intestinal *Oscillospira* species are positively correlated with secondary bile acids and negatively with primary BAs, whereas the *Bacteroidetes* phylum shows the opposite pattern (Keren et al., 2015). Interestingly, cholecystectomy increases the abundance of *Bacteroidetes*, a contributor to colorectal cancer (Keren et al., 2015). Thus, these above studies indicate that post-cholecystectomy populations are accompanied by an increased incidence of colon cancer. Consistent with previous research, our investigation showed that the genus *Bacteroides* decreased in fecal abundance in cholecystectomy patients (Figure S5), suggesting that a high level of secondary bile acids could be an important promotor of colorectal cancer incidence in cholecystectomy patients. Mass spectrometry analysis is suggested to approve what kind of secondary bile acids increase in cholecystectomy patients.

Sulfation can detoxify bile acid, with some genera of *Clostridium, Fusobacterium, Peptococcus*, and *Pseudomonas* having the capacity to desulfate sulfonated bile acids (Gérard, 2013). Our sequencing data indicated that the abundances of these bacteria were not elevated, suggesting desulfating-induced toxicity of bile acids may not be the main reason for the high risk of colorectal cancer in cholecystectomy patients. Studies have shown butyrate to be negatively correlated with colorectal cancer (Clausen et al., 1991; Bingham et al., 2003) because butyrate can act as a suppressive factor of colorectal cancer by inducing differentiation and apoptosis and inhibiting cell proliferation (Hague et al., 1995; Gonçalves et al., 2011). Furthermore, butyrate-producing *Eubacterium rectale* and *Roseburia* spp. are found at lower abundances in colorectal cancer patients (Wang et al., 2012). Even though the abundances of these bacterial genera were changed in the current study, butyrate-producing bacteria could still be considered targets for preventing and treating cholecystectomy-related colon cancer. Therefore, our study suggests performing an animal model of cholecystectomy combining butyrate-producing bacteria and toxin-producing bacteria.

## Author contributions

WW, JW, JL, QG, and JG: study design and supervision, sample handling, data analysis, and manuscript writing. PY, RZ, and WY: sample collection and carrying out of experiments. PY and YJ: collection of samples and patient information.

### Conflict of interest statement

The authors declare that the research was conducted in the absence of any commercial or financial relationships that could be construed as a potential conflict of interest.

## Supplementary material

Figure S1 Genus co-network plots based on fecal abundance of bacteria in the healthy population at different ages. Size of each pie correlates to the mean abundance of each genus across all samples. H1, 20–29; H2, 30–39; H3, 40–49; H4, 50–59; H5, over 60 years old.

Figure S2 Network plots based on fecal abundance of bacteria in the healthy population at different ages at the phylum **(A)** and genus **(B)** level. Size of each pie correlates to the mean abundance of each genus across all samples. H1, 20–29; H2, 30–39; H3, 40–49; H4, 50–59; H5, over 60 years old.

Figure S3 KEGG Bray-Curtis tree illustrating aging-associated variation of intestinal microbial function in the healthy population. H1, 20–29; H2, 30–39; H3, 40–49; H4, 50–59; H5, over 60 years old.

Figure S4 The number of OTUs identified by high-quality 16S ribosomal RNA sequencing in fecal samples from healthy volunteers and cholecystectomy patients.

Figure S5 Taxonomic classification of fecal microbiota from phylum to species in healthy volunteers and cholecystectomy patients.

Figure S6 Genus co-network plots based on fecal abundance of bacteria in cholecystectomy patients at different time stage. Size of each pie correlates to the mean abundance of each genus across all samples. D1, 5–9; D2, 10–14; D3, over 15 years after cholecystectomy.

Figure S7 KEGG Bray-Curtis tree showing post-cholecystectomy time-correlated intestinal microbial function. D1, 5–9; D2, 10–14; D3, over 15 years after cholecystectomy.

Table S1 **A** (up), **B** (down) Significant age-varied microbial KEGG functions of each age group compared with the H5 group in a healthy population (H1, 20–29; H2, 30–39; H3, 40–49; H4, 50–59; H5, over 60 years old). Difference between means was calculated by H5 group mean minus H1 group mean.

Table S2 Significantly different microbial KEGG functions between the healthy population and cholecystectomy patients. Difference between means was calculated by healthy group mean minus cholecystectomy group mean.

Table S3 Significantly different microbial KEGG functions of each post-cholecystectomy group compared with the D3 group (D1, 5–9; D2, 10–14; D3, more than 15 years after cholecystectomy). Difference between means was calculated by D3 group mean minus younger group mean.

Table S4 Cholecystectomy patient information. The population of male (M) or female (F), different age, different year range post-cholecystectomy, vegetarians, smoker, and alcohol drinker in this study.
