# Supplementary figures and images for: Cholecystectomy Damages Aging-Associated Intestinal Microbiota Construction
Source: Front Microbiol. 2018 Jun 25;9:1402. doi: 10.3389/fmicb.2018.01402 (PMC6026649; doi:10.3389/fmicb.2018.01402)

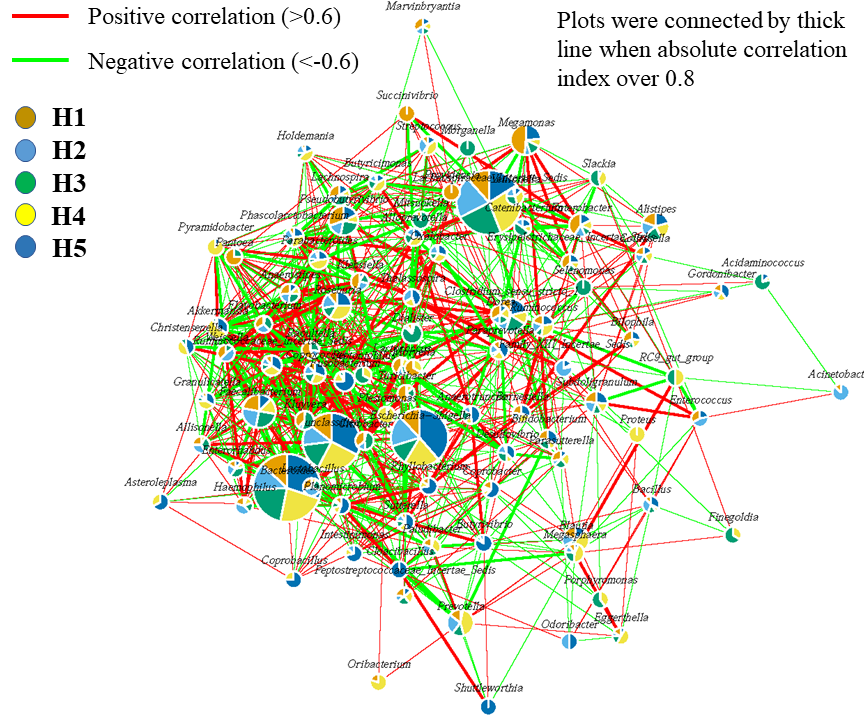

Supplement: Figure S1 — Genus co-network plots based on fecal abundance of bacteria in the healthy population at different ages. Size of each pie correlates to the mean abundance of each genus across all samples. H1, 20–29; H2, 30–39; H3, 40–49; H4, 50–59; H5, over 60 years old. [file Image_1.TIF]

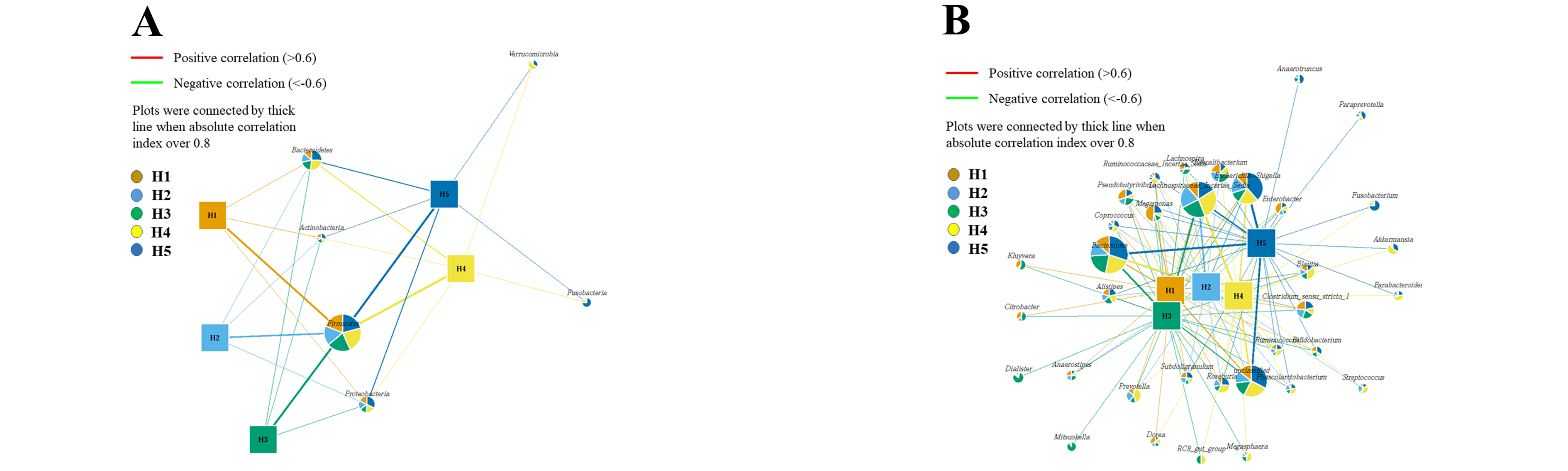

Supplement: Figure S2 — Network plots based on fecal abundance of bacteria in the healthy population at different ages at the phylum (A) and genus (B) level. Size of each pie correlates to the mean abundance of each genus across all samples. H1, 20–29; H2, 30–39; H3, 40–49; H4, 50–59; H5, over 60 years old. [file Image_2.TIF]

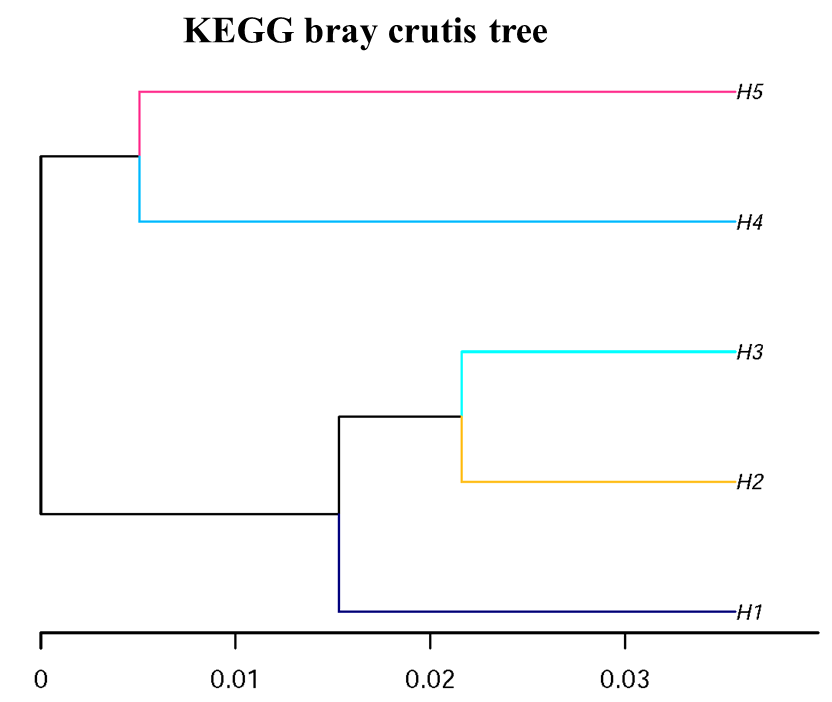

Supplement: Figure S3 — KEGG Bray-Curtis tree illustrating aging-associated variation of intestinal microbial function in the healthy population. H1, 20–29; H2, 30–39; H3, 40–49; H4, 50–59; H5, over 60 years old. [file Image_3.TIF]

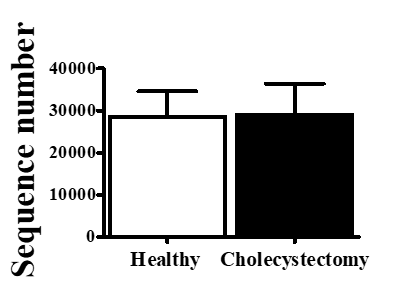

Supplement: Figure S4 — The number of OTUs identified by high-quality 16S ribosomal RNA sequencing in fecal samples from healthy volunteers and cholecystectomy patients. [file Image_4.TIF]

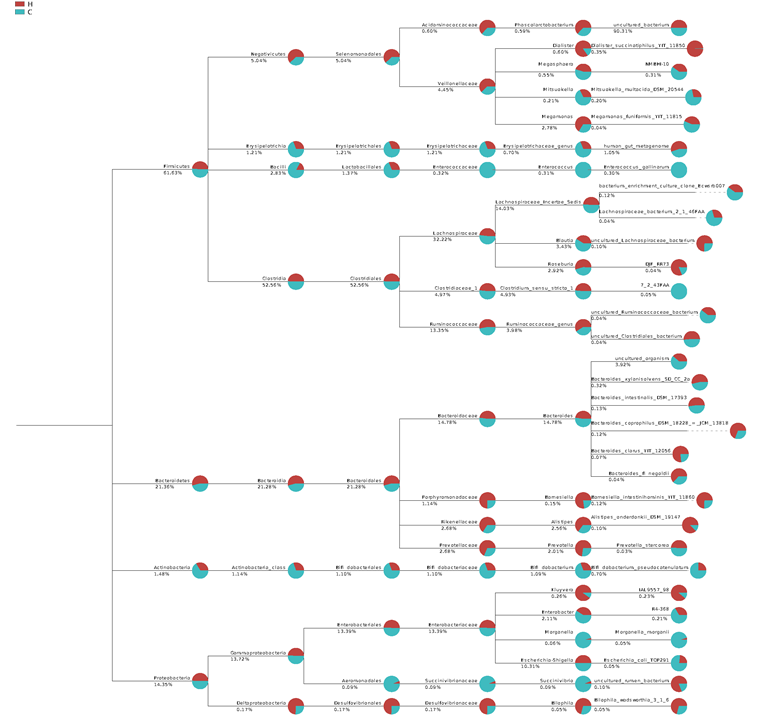

Supplement: Figure S5 — Taxonomic classification of fecal microbiota from phylum to species in healthy volunteers and cholecystectomy patients. [file Image_5.TIF]

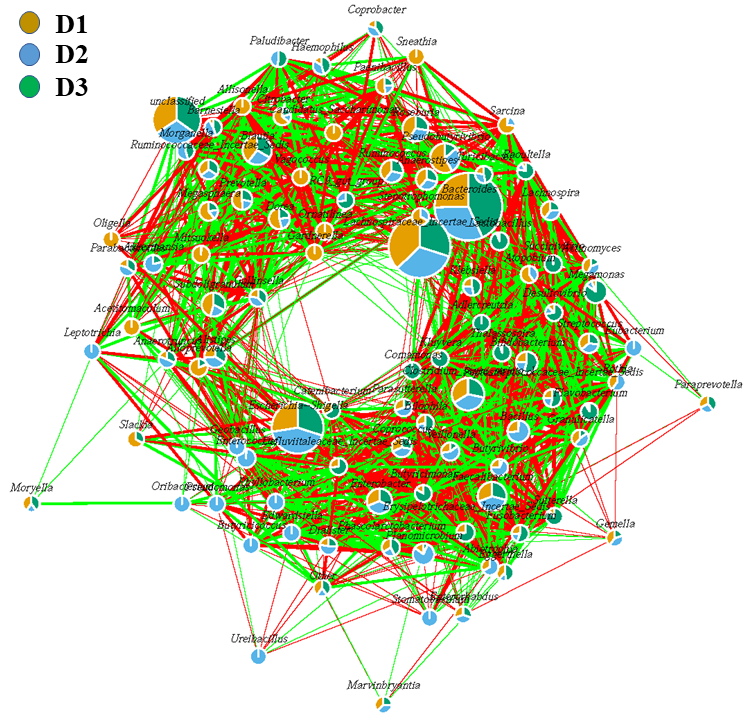

Supplement: Figure S6 — Genus co-network plots based on fecal abundance of bacteria in cholecystectomy patients at different time stage. Size of each pie correlates to the mean abundance of each genus across all samples. D1, 5–9; D2, 10–14; D3, over 15 years after cholecystectomy. [file Image_6.TIF]

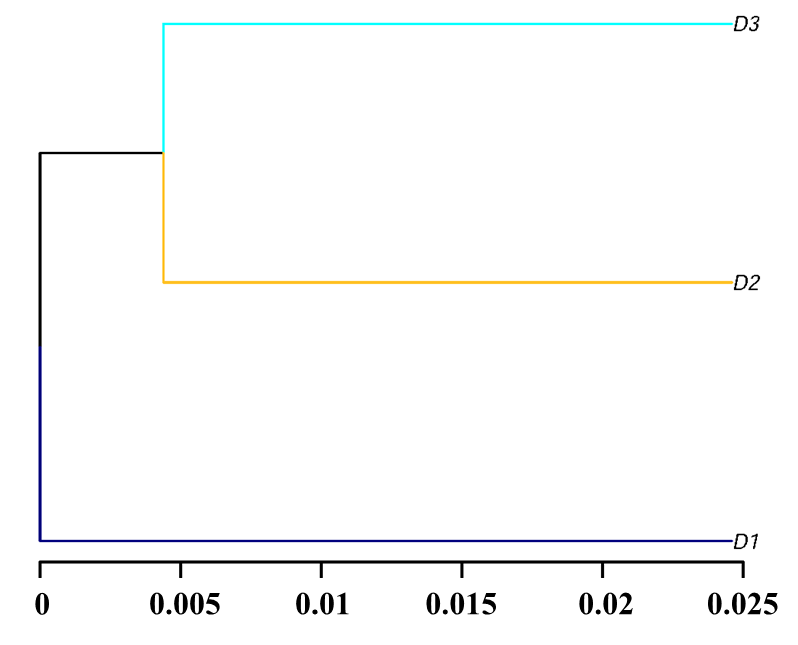

Supplement: Figure S7 — KEGG Bray-Curtis tree showing post-cholecystectomy time-correlated intestinal microbial function. D1, 5–9; D2, 10–14; D3, over 15 years after cholecystectomy. [file Image_7.TIF]
