# Supplementary material for: Cholecystectomy Damages Aging-Associated Intestinal Microbiota Construction
Source: Front Microbiol. 2018 Jun 25;9:1402. doi: 10.3389/fmicb.2018.01402 (PMC6026649; doi:10.3389/fmicb.2018.01402)
Supplement: Table S1 — A (up), B (down) Significant age-varied microbial KEGG functions of each age group compared with the H5 group in a healthy population (H1, 20–29; H2, 30–39; H3, 40–49; H4, 50–59; H5, over 60 years old). Difference between means was calculated by H5 group mean minus H1 group mean. [file Table_1.docx]

**Table S1**. **A(up)**, **B(down)** Significant age-varied microbial KEGG functions of each age group compared with the H5 group in a healthy population (H1, 20–29; H2, 30–39; H3, 40–49; H4, 50–59; H5, over 60 years old). Difference between means was calculated by H5 group mean minus H1 group mean.

**
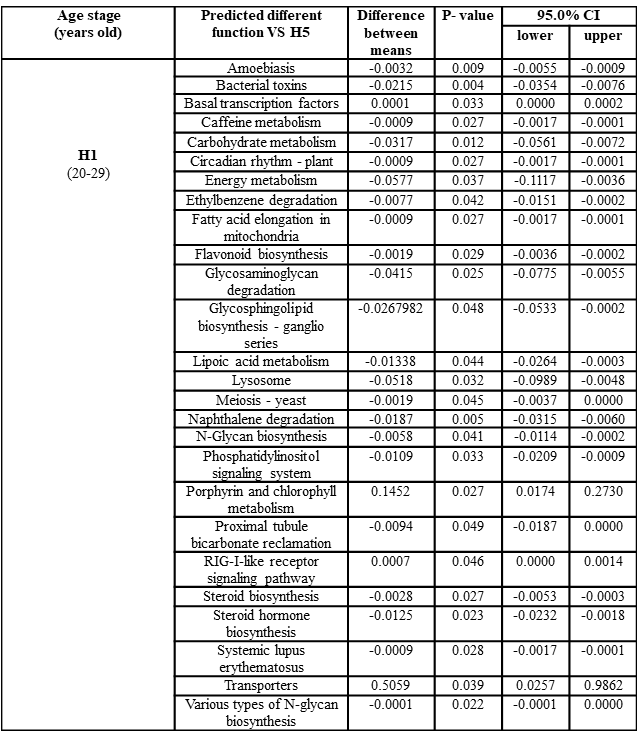
A**

**
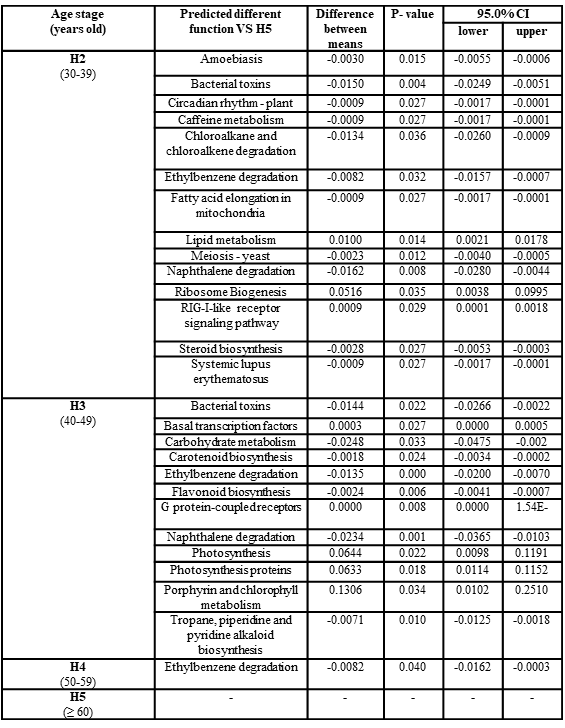
B**

**Table S2**. Significantly different microbial KEGG functions between the healthy population and cholecystectomy patients. Difference between means was calculated by healthy group mean minus

cholecystectomy group mean.

**
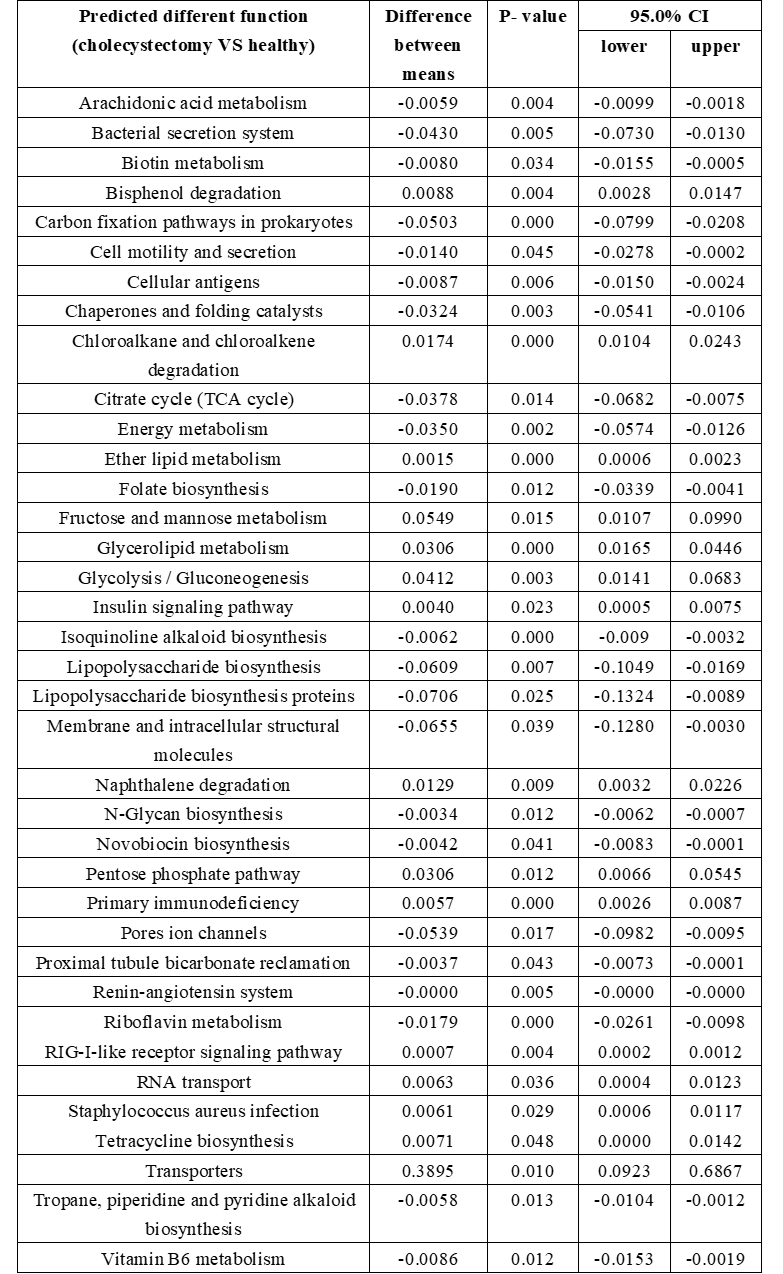
**

**Table S3**. Significantly different microbial KEGG functions of each

post-cholecystectomy group compared with the D3 group (D1, 5–9; D2, 10–14; D3, more than 15 years after cholecystectomy). Difference between means was calculated by D3 group mean minus younger group mean.


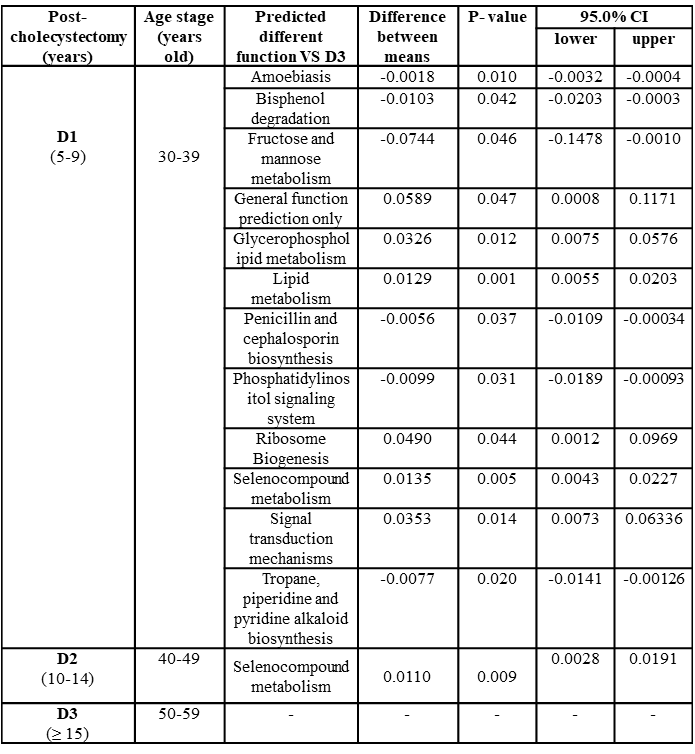


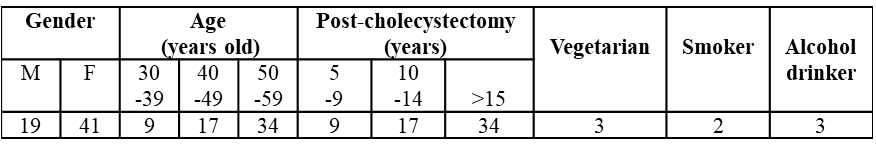
**Table S4**. **Cholecystectomy patient information**. The population of male (M) or female (F), different age, different year range post-cholecystectomy, vegetarians, smoker and alcohol drinker in this study.
